# Critical Feeding Periods for Last Instar Nymphal and Pharate Adults of the Whiteflies, *Trialeurodes vaporariorum* and *Bemisia tabaci*

**DOI:** 10.1673/031.007.3301

**Published:** 2007-05-22

**Authors:** Dale B. Gelman, Jing S. Hu

**Affiliations:** Insect Biocontrol Laboratory, USDA, ARS, PSI, Beltsville, MD 20705, USA

**Keywords:** whitefly, feeding, eclosion, fecundity, longevity

## Abstract

A critical feeding period is the time after which 50% of a given species of insect can be removed from its food source and complete development by undergoing adult eclosion. The critical feeding period was determined for the greenhouse white fly, *Trialeurodes vaporariorum*, and the sweet potato whitefly, *Bemisia tabaci* (Biotype B) (Homptera/Hemiptera: Aleyrodidae). Fourth (last) instar and pharate adult whiteflies were removed from green bean leaves, staged, placed on filter paper in small Petri dishes containing drops of water, and observed daily for eclosion. For *T. vaporariorum* reared at 25°C and L:D 16:8, 55 and 80% adult eclosion were observed when whiteflies were removed at stages 4 (0.23–0.26 mm in body depth) and 5 (≥ 0.27 mm in body depth), respectively, so that at least 50% eclosion was only achieved in this species of whitefly when adult eye development had already been initiated (in Stage 4), and 80% eclosion when adult wing development had been initiated (Stage 5). In contrast, 63% *of B. tabaci* emerged as adults if removed from the leaf at Stage 3 (0.18–0.22 mm in body depth), and 80% emerged if removed at Stage 4/5, stages in which adult formation had not yet been initiated. The mean number of eggs laid by experimental (those removed at Stages 4–5, 6–7 or 8–9) and control (those that remained on the leaf prior to eclosion) whiteflies, and the mean percent hatch of these eggs were not significantly different in experimental and control groups. Stages 7, 8 and 9 are characterized by a light red adult eye, medium red bipartite adult eye and dark red or red-black bipartite adult eye, respectively. Mean adult longevity also was not significantly different between experimental and control groups. However, for all groups of *T. vaporariorum*, adult female longevity was significantly (at least 2 times) greater than male longevity. Our results identify the critical feeding periods for last instar/pharate adults of two important pest species of whitefly. Since in both species of whitefly the critical feeding period is achieved when weight gain reaches a plateau, it appears that the critical feeding period is more closely correlated with the attainment of a critical weight than with either the time that ecdsyteroid titers first peak or the time when adult development is initiated.

## Introduction

The sweet potato whitefly, *Bemisia tabaci* Biotype B [also known as the silverleaf whitefly, *Bemisia argentifolii* (Bellows et al., 1994)], attacks more than 500 different species of plants in both field and greenhouse settings, including food, fiber and ornamental species. The greenhouse whitefly *Trialeurodes vaporariorum* is also a polyphagous species and is primarily a serious pest of plants grown in greenhouses. World-wide, whiteflies cause billions of dollars of damage in crop losses each year by feeding on plant phloem, transmitting plant pathogenic viruses (primarily *B. tabaci*) and producing honeydew that causes stickiness and supports the growth of sooty mold (Perkins and Bassett, 1988; Gill, 1992; Zalom et al., 1995; Heinz, 1996; Henneberry et al., 1997; Henneberry et al., 1998; Chu and Henneberry, 1998). However, although there is a growing body of literature concerning the basic biology of whiteflies and the physiology of feeding, the critical feeding period, the time (stage) after which 50 percent of the whiteflies can be removed from the leaf and complete development, i.e., emerge as adults, has not been investigated. Precise staging systems both for identifying instar and for tracking developmental progress of 4th instar/pharate adult whiteflies grown on green bean and other plants have been described (Gelman et al., 2002a; b; Gelman and Gerling, 2003). Since for each whitefly instar, body depth and not length or width increases (Hargreaves, 1915), and in 4th instars, body depth reaches a maximum just before the initiation of adult formation, 4th instar nymphal stages were assigned based on body depth. Briefly, Stages 1, 2 and 3 were characterized by body depths of 0.1, 0.15. and 0.2 ± 0.02 mm, respectively; stage 4 had a body depth of 0.23–0.26 mm, and nymphs with a body depth ≥0.27 mm were assigned to Stage 5. Stages 6 through 9 were identified based on the appearance of the developing adult eye. Nymphs entered Stage 6 when the small intense red dot characteristic of the eye of Stages 1 through 5 began to diffuse. Stages 7, 8 and 9 were characterized by a light red, medium red bipartite, and dark red or red-black bipartite adult eye, respectively. In *T. vaporariorum*, adult eye development is initiated in Stage 4 and adult wing development in Stage 5, before the diffusion of the red dot is visible in whiteflies examined under a stereoscopic dissecting microscope, while *B. tabaci* initiates adult development at Stage 6 (Gelman et al., 2002a; 2002b). Here, we report that even though whiteflies feed through Stage 9 (Lie et al., 1996; Costa et al., 1999; Gelman et al., unpublished results), the stage that precedes adult eclosion, at least 50 percent of *B. tabaci* can be removed from the leaf prior to the initiation of adult development, and at least 50 percent of *T. vaporariorum* removed when adult eye formation is initiated, and these whiteflies will go on to complete adult development, emerge, mate and lay viable eggs.

## Materials and Methods

### Insect Rearing

Whiteflies were reared on a variety of plants to maintain whitefly diversity. They were grown on green bean cv. Roma II (Burpee, Warminster, PA, USA), tomato cv. Bush Big Boy (Burpee, Warminster, PA, USA), cotton cv. Stoneville ST 474 (Stoneville Pedigreed Seed Co., Maricopa, AZ, USA), poinsettia cv. Freedom Red (Paul Ecke Ranch, Encinitas, CA, USA) and eggplant cv. Millionaire Hybrid (Burpee, Warminster, PA, USA). All of the plants except for poinsettia [grown from cuttings supplied by Paul Ecke Ranch (Encinitas, CA, USA)] were grown from seed as described in Gelman et al. (2002a; b). A walk-in, climate-controlled insect growth chamber (26 ± 2°C, L:D 16:8 and relative humidity of 60–80%) was used to house the B. *tabaci* colony, while the *T. vaporariorum* colony was maintained in a greenhouse under similar environmental conditions at 26 ± 4°C. Green bean plants were infested by placing greenhouse-grown plants into the *B. tabaci* or *T. vaporariorum* colonies and removing the plants 24 h later. Almost all of the adult whiteflies were removed, and the infested plants were transferred to a second growth chamber or to an incubator box that provided the same rearing conditions as described previously for the colonies. *T. Vaporariorum* and *B. tabaci*-infested plants were maintained in separate chambers.

### Insect staging

*T. vaporariorum* and *B. tabaci* 4th instar/pharate adults were assigned to stages as described elsewhere (Gelman et al., 2002a; b). An optical micrometer mounted on a stereoscopic microscope was used to identify 4 instar nymphs (0.6–0.8 mm in length) and to distinguish among the nymphal stages of both *T. Vaporariorum* and *B. tabaci.* Whiteflies from more than fifty different green bean plants were sampled for each of the two species of whitefly.

**Figure 1.**
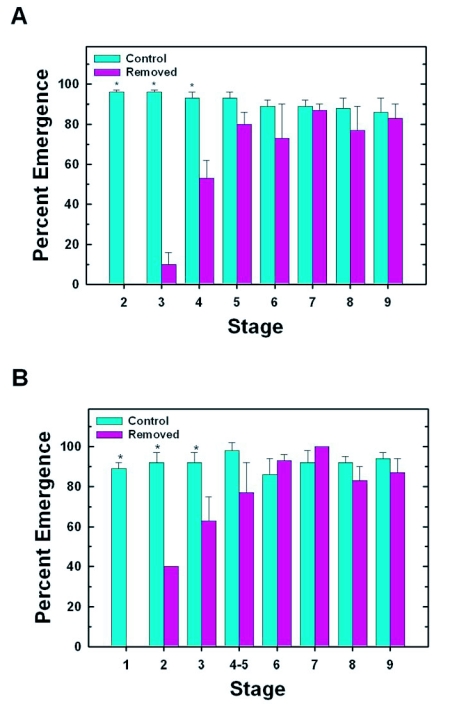
Effect of stage of whitefly removal on percent *T. vaporariorum* (A) and *B. tabaci* (B) adult eclosion. Experimental *T. vaporariorum* or *B. tabaci* were removed from green bean leaves at the stage indicated and placed on moist filter paper. Control whiteflies (whiteflies that completed their life cycle on the leaf) were staged and their positions on the leaf were mapped. Adult eclosion for individual whiteflies in each stage was recorded. Each value represents the mean ± S.E. of at least four separate replicates; for each replicate a minimum of 20 whiteflies was examined. S. E. bars not shown were smaller than the size of the datum point. For Stage-2 experimental *T. Vaporariorum* (Fig. 1B), 0% emerged. For each stage, the Student's t-Test (α = 0.05) was used to determine if there was a significant difference between the experimental group (pink bar) and its respective control (blue bar). An asterisk indicates that there is a significant difference between the two groups.

### Effect of stage of whitefly removal on percent adult eclosion

Fourth instar *T. vaporariorum* (stages 1–5) or *B. tabaci* nymphs (Stages 1–4/5) [the ratio of Stage 4: Stage 5 was approximately 8:1 probably because many *B. tabaci* proceed directly from Stage 4 to Stage 6 (Gelman and Gerling, 2003)] and pharate adults (Stages 6–9) were carefully removed from the undersurface of green bean leaves, the stage of each whitefly was determined, and whiteflies of the same stage were placed on small pieces of filter paper in 10 × 35 mm Petri dishes. To maintain high humidity, small drops of water were placed around the filter paper at the edges of the dish, and each small Petri dish was placed in a 20 × 100 mm Petri dish containing moist filter paper. Whiteflies were examined daily for eclosion. Experiments were designed so that percent eclosion for each experimental stage could be compared with percent eclosion for the control group of the same stage. For controls, the undersurfaces of infested leaves that were in close proximity to the leaves from which experimental whiteflies were selected and removed, were examined under the microscope. A small pen mark was placed next to 4th instars (selected at random) and a map indicating the location of each selected whitefly nymph was generated. Since control whiteflies could not be removed from the leaf, the stage of these whitefly nymphs was assigned based on an estimate of body depth. Exceedingly flat whiteflies were designated as Stage 1, slightly raised whiteflies as Stage 2 and thicker whiteflies as Stages 3, 4 or 5. While the accuracy of stage assignments for controls as compared to experimental whiteflies was, of course, reduced, in experiments in which stage was estimated, our estimate was accurate approximately 85 percent of the time. When we erred, it was only by one stage, and this typically occurred when distinguishing between Stage-3 and -4 whiteflies. For both the experimental and the control groups, for each of the four to six replicates of each stage, whiteflies from two separate plants were sampled.

### Determination of 4th instar/pharate adult weights of *B. tabaci* and *T. vaporariorum*

Body weights were measured using a Cahn Model C-34 microbalance (ATI Orion, orionres.com). Individual whiteflies were carefully removed from leaves of 3–4 green bean plants and the stage of each nymph or pharate adult was determined. For each replicate, 2–3 nymphs/pharate adults of the same stage were weighed and the mean weight/individual whitefly was calculated. 10–12 replications were performed for each stage of each species of whitefly.

### Statistical Analysis

When means of three or more groups were compared, a one-way ANOVA followed by the Tukey's HSD comparison of means test was used to determine if means were significantly different (α = 0.05) from each other. The Student's t-Test was used when comparing an experimental group with its respective control group (α = 0.05).

### Effect of stage of removal on number of eggs laid, percent egg hatch and longevity of adults

For each experimental or control group, individual whiteflies were mated by placing a recently emerged male and female into a clip cage which was then attached to a green bean leaf with the chamber facing the undersurface of the leaf. For controls, adult whiteflies that had been reared on leaves of two to three plants (used to monitor the eclosion of control whiteflies) were collected. Adults remained in the clip cages for the duration of the experiment. All clip cages were carefully moved to new sections of the leaf every three days for the next 12 days and the number of eggs laid was determined until day 15. Leaves were examined every two days to monitor egg hatch and adult mortality until both adults had died and each egg had either hatched or had desiccated.

## Results

### Effect of stage of whitefly removal on percent adult eclosion

For both *T. vaporariorum* and *B. tabaci*, percent eclosion through Stage 5 and Stage 4/5, respectively, increased as the stage at which the whitefly was transferred from the leaf to the Petri dish increased (Figure 1A and B). Once the 4th instar nymph of either species was removed from the leaf it did not increase in body depth (results not shown). For *T. vaporariorum*, slightly more than 50% eclosion was observed at Stage 4, and by Stage 5, percent eclosion was approximately 80% and not significantly different from that of its control (Figure 1A). For *B. tabaci*, percent eclosion exceeded 50% by Stage 3 and was not significantly different from its control value, which was approximately 75% by Stage 4/5. The onset of adult development in *T. vaporariorum*, occurred in Stages 4 (eye) and 5 (wing), while in *B. tabaci*, the initiation of both adult eye and wing development occur in Stage 6.

**Figure 2.**
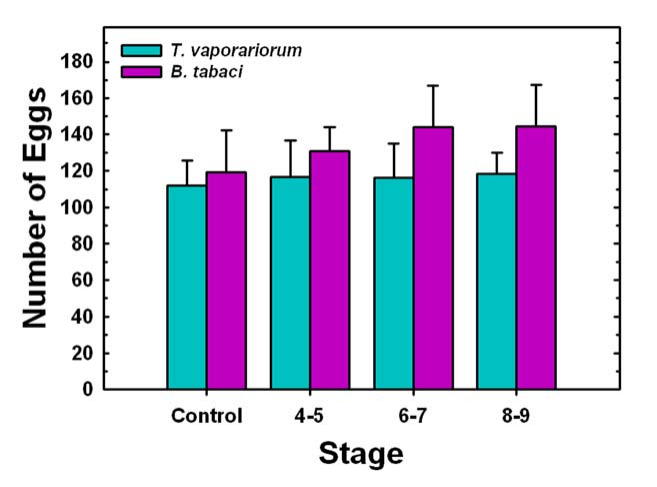
Effect of stage of removal on the number of eggs laid by adults that had emerged from experimental and control groups of whitefly nymphs. For both control and experimental groups, a young male and a young female whitefly were placed in a clip cage that was attached to a green bean leaf. The number of eggs laid was determined until day 15. For *T. vaporariorum* and *B. tabaci*, respectively, each value represents the mean ± S.E. of eggs laid by 23–40 and 20–37, separate replicates (whitefly pairs). For each species of whitefly, a one-way ANOVA (α = 0.05) was used to determine if there were any significant differences among the four groups, i.e, the control and the three experimental groups. Since for *T. vaporariorum* as well as *B. tabaci*, P was >0.05, the Tukey's comparison of means test was not performed.

**Figure 3.**
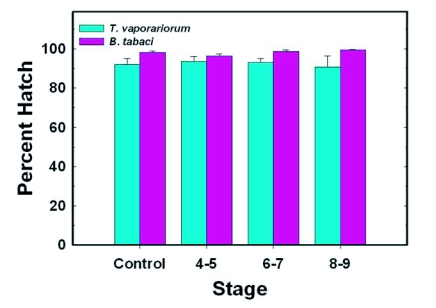
Effect of stage of removal on percent egg hatch of eggs laid by adults that had emerged from experimental and control groups of whitefly nymphs. A brief description of the methods used for mating adult control and experimental whiteflies is provided in the legend for Fig. 2. For *T. vaporariorum* and *B. tabaci*, respectively, each value represents the mean ± S.E. of 14–17 and 8–14 separate replicates/mated pairs; each replicate was based on an examination of 80–100 eggs. A one-way ANOVA (α = 0.05) was used to determine if there were significant differences among control and experimental groups for each species of whitefly. Since P was >0.05 when means for control and experimental groups of each species were compared, the Tukey's comparison of means test was not performed.

**Table 1.**
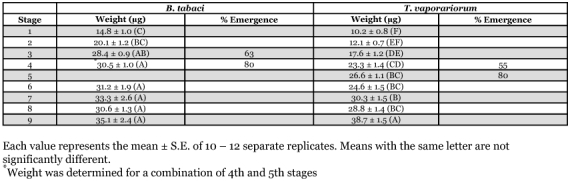
Weights of 4th instar/pharate adult *B. tabaci* and *T. vaporariorum.*

For those whiteflies that emerged as adults, the duration in days for a given stage to undergo ecdysis was not significantly different in experimental and control insects for Stages 6–9. When nymphs were removed at Stage 4 or 5, ecdysis was delayed by 1–2 days as compared to controls (result not shown), and thus, there was a significant difference in time to ecdysis for experimental and control groups of these stages.

### Weights of 4th instar/pharate adult whiteflies

The weight of each stage of the nymphs and pharate adults of the two whitefly species is provided in Table 1. For *B. tabaci*, body weight increased between Stages 1 and 3, and plateaued between Stages 3 and 9, while in *T. vaporariorum*, body weight increased between Stages 1 and 4, plateaued between Stages 4 and 8 and exhibited a significant increase in Stage 9.

### Effect of stage of removal on number of eggs laid and percent egg hatch

For both *T. vaporariorum* and *B. tabaci*, the numbers of eggs laid by whiteflies that had emerged from nymphs removed from the leaf at Stages 4–5, 6–7 or 8–9 were not significantly different from control values (Figure 2). Mean numbers of eggs laid ranged between 111–119 and 119–44 for *T. vaporariorum* and *B. tabaci*, respectively. Percent egg hatch was relatively high (>90%) for control and experimental groups of both whiteflies, and means for groups in which whiteflies were removed from the leaf at Stages 4–5, 6–7 and 8–9, were not significantly different from their respective control groups (Figure 3).

### Effect of stage of removal on adult longevity

**Figure 4.**
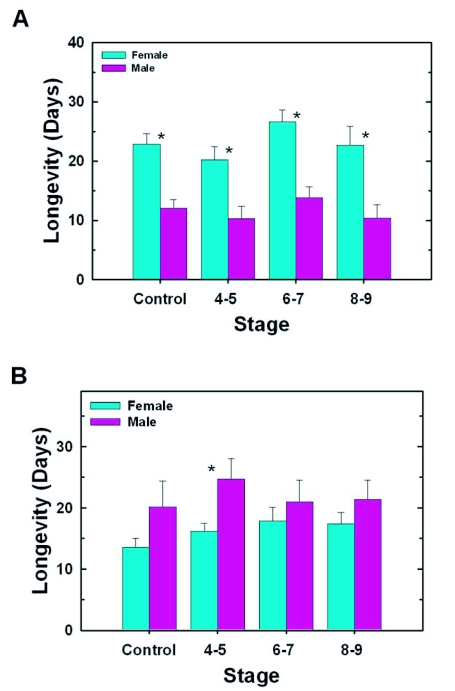
Effect of stage of removal on adult longevity of *T. vaporariorum* (A) and *B. tabaci* (B). A brief description of the methods used for mating adult control and experimental whiteflies is provided in the legend for Fig. 2. Longevity (in days) was determined for mated whiteflies. Each value represents the mean ± S.E. for 10 – 20 whiteflies. For each species and for each sex, a one-way ANOVA (α = 0.05) was used to determine if there were significant differences among control and experimental groups. Since P was >0.05 the Tukey's comparison of means test was not performed. A Student's t-Test (α = 0.05) was used to compare the means of males and females for each experimental and each control group of each species of whitefly. An asterisk indicates that there is a significant difference in longevity between the sexes.

Mean longevity for experimental groups of either female or male *T. vaporariorum* were not significantly different from each other or from their respective control groups (Figure 4A). However, for each group of experimental or control *T. vaporariorum*, female longevity was approximately twice as great as male longevity (Fig. 4A). A comparison of mean longevities for control and experimental groups of *B. tabaci* males and separately, for control and experimental groups of *B. tabaci* females, also showed that there was no significant difference between mean longevities of control and experimental groups for either sex (Figure 4B). However, in contrast to the situation for *T. vaporariorum*, in *B. tabaci*, males tended to live longer than females, and for the group in which nymphs were removed in Stage 4–5, mean longevity of adult males was significantly greater than that of adult females.

## Discussion

The effect of the stage of removal of whitefly nymphs/pharate adults from green bean leaves on the ability of *T. vaporariorum* and *B. tabaci* to complete their life cycle and emerge as adults, and on the robustness of these adults, was determined and this information was used to identify the critical feeding period for each species of whitefly. Although adult development of *T. vaporariorum* and *B. tabaci* was initiated in Stage 4(eye)/5 (wing) and 6, respectively (Gelman et al., 2002a; 2002b), the critical feeding period was achieved at an earlier stage in *B. tabaci* (between Stage 2 and 3, mean percent eclosion = 40 and 63%, respectively) than in *T. vaporariorum* (at approximately Stage 4, mean percent eclosion = 55%). In addition, no *T. vaporariorum*, but 40% of *B. tabaci* that were removed at Stage 2 were able to survive to the adult stage. Thus, to achieve 50% adult eclosion, *T. vaporariorum* requires a food source up to the stage at which adult eye development is initiated, i.e., Stage 4 (Gelman et al., 2002a), while 63% of Stage-3 *B. tabaci* completed adult development and emerged even when deprived of food at Stage 3, well before the initiation of adult development which occurs in Stage 6 (Gelman et al., 2002b). The ability of some nymphs of both whitefly species to survive when food is unavailable is noteworthy since whiteflies continue to produce honeydew through Stage 9 and, thus, in nature probably feed until, or just prior to, adult ecdysis (Lie et al., 1996; Costa et al., 1999; Gelman et al., unpublished results). The quality of the whiteflies that went on to emerge as adults was equal to that of controls in that fecundity (eggs laid and hatched), and longevity, of experimental and control groups for each species of whitefly were not significantly different. While differential fecundity, survivorship and/or mating are primarily responsible for differences in success between two competing species, the ability of many *B. tabaci* nymphs as compared to *T. vaporariorum* nymphs to complete adult development and emerge when food is unavailable at Stage 2 and 3, as well as the relatively short life span of *T. vaporariorum* males as compared to *B. tabaci* males may contribute to the greater vitality of *B. tabaci.* Thus, when a *T. vaporariorum* colony was contaminated with some *B. tabaci*, in less than 3 months after *B. tabaci* nymphs were first observed on plant leaves (approximately 2% infestation), *T. vaporariorum* was almost completely replaced by *B. tabaci* which now represented approximately 90% of the population (unpublished results).

In other insect species, the critical feeding period also is achieved before feeding is normally terminated. Thus, in the holometabolous yellow-spotted longicorn beetle, *Psacothea hilaris*, an important pest of sericulture in Japan, the critical feeding period occurs on the third day of the 4th instar, long before pupation, an event that normally occurs at the end of the 5th instar (Shintani et al., 2003). In that insect species, starvation on or after the third day of the 4th instar results in premature pupation and the production of relatively small pupae. The longer the larvae are fed, the greater the pupal weight. Based on their results, the authors concluded that size was not a proximate cue for pupation. However, there is a threshold weight that larvae must reach in order for a viable pupa to be formed (Munyiri et al., 2003). In many insects, a threshold weight must be achieved for larval-pupal or nymphal-adult metamorphosis, i.e., the occurrence of the larval-pupal molt or nymphal-adult molt [Nijhout, 1975 (*Manduca sexta*); Nijhout, 1981 (*M. sexta*); Jones et al., 1981 (*Trichoplusia ni*); Tanaka, 1981(*Blatella germanica*); Sehnal, 1971 (*Galleria mellonella*)]. In some insect species, when diet is poor additional larval molts occur so that the insect achieves the critical weight, e.g., in Lepidoptera (Williams, 1976) and *Blattaria* (Lefeuvre, 1969). In others, the number of instars preceding the metamorphic molt is constant, and the length of the instar, and thus period of feeding, is increased, e.g., in most Diptera, Hymenoptera and some beetles [as reviewed in Sehnal (1985)]. However, in either scenario, there is a critical feeding period (usually linked to weight) before which 50% of the insects will not be able to survive, metamorphose and complete their life cycle. It is noteworthy that starvation may reprogram the insect by causing a premature hormonal change such as a rapid decline in juvenile hormone titer that, in turn, cues a small ecdysteroid peak that results in a premature commitment to metamorphosis (Munyiri and Ishikawa, 2005).

In *B. tabaci*, ecdysteroid concentration peaks between Stages 4 and 6 and adult development is initiated in Stage 6 (Gelman et al., 2002b). In *T. vaporariorum*, ecdysteroid titers peak between Stages 3 and 6 and adult eye and wing development are initiated in Stages 4 and 5, respectively (Gelman et al., 2002a). Thus, in *T. Vaporariorum*, both the stage in which the ecdysteroid titer first peaks (Stage 3) and the stage in which adult development is initiated (Stage 4/5) are at least one stage earlier than the stages in which these events occur in *B. tabaci* (Stages 4/5 and 6, respectively). Yet the critical feeding period is reached one stage earlier in *B. tabaci* (Stage 3) than in *T. vaporariorum* (Stage 4).

Based on the determination of fluctuations in weight during the 4 stadium (Table 1), it appears that the critical feeding period is linked to the attainment of a critical weight rather than to either the timing of the ecdysteroid peak or the initiation of adult development. Once the 4^th^ instar of either whitefly was removed from the leaf and placed on filter paper, it did not increase in body depth. On the leaf, *B. tabaci* 4th instars increase in weight until Stage 3 and then reach a plateau through Stage 8, while *T. vaporariorum* increase in weight through Stage 4 and then reach a plateau. Stages 3 and 4, respectively, are the stages in which the critical feeding period was observed in *B. tabaci* and *T. vaporariorum.* And, if in whiteflies, a critical weight is also required to trigger an increase in ecdysteroid, which, in turn, is responsible for initiating the nymphal-adult molt, the critical weight is lower in *T. vaporariorum* than in *B. tabaci.*
